# Lymphocyte and CD62E expression in lichen planus and lichenoid reaction

**DOI:** 10.1186/s12903-022-02496-5

**Published:** 2022-11-17

**Authors:** Juliana Tristão Werneck, Lúcio Souza Gonçalves, Letícia Côgo Marques, Arley Silva Junior

**Affiliations:** 1 Instituto de Saúde de Nova Friburgo – Universidade Federal Fluminense (UFF), Nova Friburgo, RJ Brazil; 2 Postgraduation Program in Dentistry, Faculty of Dentistry, Universidade Estácio de Sá, Rio de Janeiro, RJ Brazil; 3 Department of Pathology, School of Medicine, Universidade Federal Fluminense, Niterói, RJ Brazil

**Keywords:** Oral lichen planus, Oral lichenoid reaction, Lymphocytes, CLA, E-selectin

## Abstract

**Background:**

It is difficult to distinguish the clinical and histopathological aspects of oral lichen planus lesions from those of oral lichenoid reaction. Some criteria were proposed to distinguish them, mainly because they have different biological behaviors. The aim of the present study was to compare the lymphocyte population and the expression of E-selectin between these lesions.

**Methods:**

Participants with a clinical diagnosis of oral lichen planus (GOLP) and oral lichenoid reaction (GOLR) who needed to perform a biopsy were selected. The tissue was frozen and immunostaining was performed for CD3/CD4, CD3/CD8, CD4/CLA, CD8/CLA, and CD62E. The analysis of each immunostaining was accomplished using the ImageJ program.

**Results:**

In total, 25 participants with oral lichen planus and 11 with oral lichenoid reaction were seen. In the evaluation of CD3 + CD4+/CD3 + and CD3 + CD8+/CD3 + proportions, there was a higher percentage of these cells in the oral lichen planus group when compared with the oral lichenoid reaction group (p = 0.027 and p = 0.038 respectively). The average number of CLA + lymphocytes for CD4+/CLA + and CD8+/CLA + in both groups was not statistically significant (p = 0.840; d = 0.363). In GOLP, the number of CD4 + CLA+/E-selectin and CD8 + CLA+/E-selectin was not statistically significant (p = 0.951 and p = 0.454 respectively); neither in GOLR (p = 0.454 and p = 0.989 respectively).

**Conclusion:**

Our results indicate that CD3 + CD4+, CD3 + CD8+, CD4 + CLA+, CD8 + CLA + lymphocytes and E-selectin are present in both lesions. However, the proportion of CD3 + CD4+/CD3 + and CD3 + CD8/CD3 + cells is higher in the oral lichen planus group when compared with the oral lichenoid reaction group, suggesting that these cells may be important for the etiopathogenic mechanism of these lesions.

**Supplementary Information:**

The online version contains supplementary material available at 10.1186/s12903-022-02496-5.

## Background

Lichen planus is a mucocutaneous disease that can be present only in the oral mucosa in a polymorphic form [1–3]. One of the most discussed issues today is associated with the diagnosis of oral lichen planus (OLP). The main reason for defining diagnostic criteria is based on the potential for the malignant transformation of these lesions, as it is unknown whether OLP undergoes malignant transformation [3, 4]. Oral lichenoid reactions (OLR) may not differ in clinical and histopathological aspects from classic lesions of OLP [4, 5]. In case of suspicion, an association of the lesion with the use of systemic medication and restorative materials should be verified [5, 6].

OLR may be a delayed hypersensitivity reaction, in which helper CD4 T and cytotoxic CD8 T lymphocytes act by releasing cytokines (TNF-α and IFN-δ), which activate pro-inflammatory cells resulting in tissue damage [7, 8]. Conversely, in the case of OLP, the cytotoxic CD8 T lymphocytes, activated by the helper CD4 T lymphocytes, lead keratinocytes to apoptosis, cause the disruption of the basal membrane and the entry of lymphocytes into the epithelium. These cells release RANTES chemokine and TNF-α. This cytokine will activate E-selectin in blood vessels, which is an adhesion molecule for lymphocyte migration and the main adhesion molecule for migration of cutaneous lymphocyte-associated antigens (CLA+) [8–13]. CLA represent a subpopulation of lymphocytes present in abundance in inflamed areas of the skin, but it can be found in the oral mucosa and normal skin [14–19].

Thus, the aim of this study was to compare lymphocyte populations and the expression of E-selectin in lesions of oral lichen planus with the oral lichenoid reaction.

## Methods

This study was approved by the Research Ethics Committee of Hospital Universitario Antonio Pedro (CAAE: 47567515.1.0000.5243). Participants who presented lesions in the oral mucosa compatible with OLP or OLR, without corticoid treatment, and who needed to perform biopsies were selected from a period between 2008 and 2017. Inclusion criteria for oral lichen planus group (GOLP) were based on van der Meij & van der Waal [3], where those who clinically presented bilateral reticular lesions and/or other patterns of OLP associated with the reticular pattern; and who histopathologically on hematoxylin and eosin stain presented hydropic degeneration of the basal cell layer, predominantly infiltrating lymphocytes, in band, confined to the upper part of the connective tissue, and absence of epithelial dysplasia were included in this group. The group of oral lichenoid reaction (GOLR) included participants who did not meet one or more clinical or histopathological criteria for OLP. Biopsies were performed in the reticular pattern and in the buccal mucosa.

**Fig. 1:**
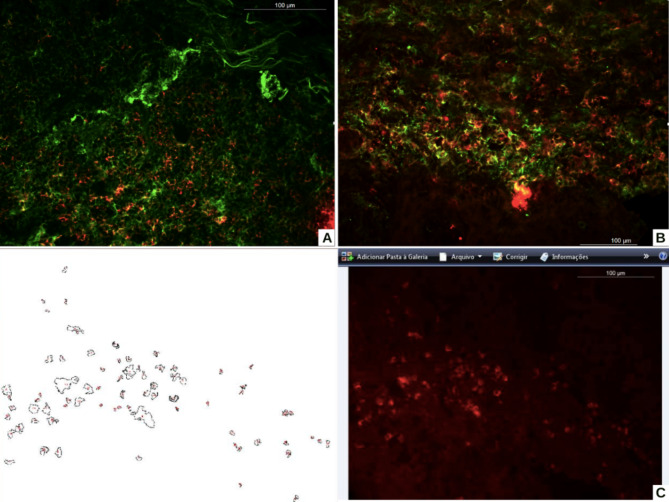
Comparison between groups regarding to CD3 + CD8 + cells (FITC for CD3 and Texas red for CD8 – double immunofluorescence staining in yellow): oral lichen planus (A); oral lichenoid lesion (B); Exemplification of the cell counting process (Texas red for CD8) using the Image J program (C)

All obtained fragments were immediately included in OCT and frozen in the − 80 °C freezer. Subsequently, each glass slide received three fragments of 6-µm cuts of the specimens. Double immunofluorescence staining was performed for CD3/CD4, CD3/CD8, CD4/CLA, CD8/CLA, and for E-selectin (CD62E) (Table 1) (Fig. 1, A and B). Each immunofluorescence staining was photographed with a 40X objective in five hot spots, totaling a minimum of 150 cells. Cell counting was performed using the ImageJ program, and the counting of vessels labeling for E-selectin was manually counted (Fig. 1, C).

**Table 1:** Antibodies and dilutions used in immunofluorescence

Antibodies	Dilution for FITC	Dilution for Texas Red Dye	Supplier
**CD3**	1:200	1:200	DAKO, Santa Barbara, CA
**CD4**	1:200	1:300	DAKO, Santa Barbara, CA
**CD8**	1:300	1:100	DAKO, Santa Barbara, CA
**CD62E**	1:100	1:100	Becton, Dickinson & Co; San Diego, CA
**CLA**	**1:50**	**–**	Becton, Dickinson & Co; San Diego, CA

All analyses were performed using the Statistical Package for the Social Sciences (SPSS) program, version 21.0 (IBM, Armonk, NY, USA). The normality of continuous variables was verified using the Kolmogorov-Smirnov and Shapiro-Wilk tests in addition to graphical analyses. In the comparative analyses between the two groups, Student’s t-test was used for variables with normal distribution (CD3 + CD4+, CD4 + CLA+, CD4 + CLA+/CD4+, CD8 + CLA+/CD8+, CLA+); and the Mann-Whitney test, for variables with non-normal distribution (CD3 + CD4+/CD3+, CD3 + CD8+, CD3 + CD8+/CD3+, CLA+/CD4+, CD8 + CLA+, CLA+/CD8+, CD62E). Pearson’s correlation coefficient was estimated to measure the statistical relationship between CD4 + CLA + and E-selectin, and CD8 + CLA + and E-selectin. As the previous sample calculation was not performed, the power of the statistical tests used for each variable was calculated by using the GPower 3.1.9.2 software. The power of a test depends on three factors: effect size, significance level, and sample sizes [20]. Thus, after applying the statistical tests in the comparison between groups (obtaining the level of significance for each analysis), the effect size was calculated for each variable [21]. The following interpretation criteria were used for the effect size (d): no effect (d ≤ 0.1), small (0.20 ≤ d ≤ 0.40), medium (0.50 ≤ d ≤ 0.70), and large (d ≥ 0.80) [21].The effect size estimated the magnitude of the difference between groups. The established level of statistical significance was 5% (p ≤ 0.05) for all analyses.

## Results

In total, 36 participants were seen; of these, 25 (69.5%) had histopathological diagnosis for oral lichen planus and 11 (30.5%) for oral lichenoid reaction.

Of the 36 participants, three (8.3%) were men, two of the GOLP and one of the GOLR. Among GOLP participants, age ranged between 24 and 79 years (mean of 55.8 years). Among GOLR participants, age ranged between 46 and 82 years (mean of 67.4 years).

The comparison between the number of CD3 + CD4+ (15,678, in total) and CD3 + CD8+ (18,983, in total) lymphocytes in GOLP was performed and the difference was statistically significant (p < 0.001; d = 0.353). The analysis was also performed between CD4 + CLA+ (9,594, in total) and CD8 + CLA+ (7,856, in total) lymphocytes within the same group, and the difference was also statistically significant (p = 0.031; d = 0.373).

Likewise, we compared the number of CD3 + CD4+ (6,510, in total) and CD3 + CD8+ (5,918, in total) lymphocytes in GOLR and the difference was not statistically significant (p = 0.154; d = 0.214). The analysis between CD4 + CLA+ (4,954, in total) and CD8 + CLA+ (4,908, in total) lymphocytes within the same group was not statistically significant as well (p = 0.082; d = 0.02).

In Table 2, it is observed the comparisons between both groups with regard immunofluorescence analyses for CD3, CD4, CD8, CLA, and E-selectin. The comparison of the number of CD3 + CD4+ (p = 0.615; d = 0.127) and CD3 + CD8+ (p = 0.099; d = 0.571) lymphocytes in both groups was not statistically significant. However, the proportion of CD3 + CD4 + lymphocytes in relation to CD3 + lymphocytes in both groups was statistically significant (p = 0.027; d = 0.794). In addition, the proportion of CD3 + CD8 + lymphocytes in relation to CD3 + lymphocytes (p = 0.038; d = 0.738) was also statistically significant.

**Table 2:** Results of the immunofluorescence analysis for CD3, CD4, CD8, CLA, and E-selectin comparing GOLP and GORL

Immunostaining	Total number of lymphocytes		***p*** ^*^	***d***
	**GOLP**	**GORL**		
**CD3+** ^**§**^	25,832	13,469	0.311	0.343
**CD3 + CD4+** ^**¥**^	15,678	6,510	0.615	0.127
**CD3 + CD4+/CD3+** ^**§**^	68,74	54,45	**0.027**	0.794
**CD3 + CD8+** ^**§**^	18,983	5,918	0.099	0.571
**CD3 + CD8+/CD3+** ^**§**^	67,91	51,74	**0.038**	0.738
**CLA+** ^**¥**^	16,176	8,296	0.840	0.363
**CD4 + CLA+** ^**¥**^	9,594	4,954	0.888	0.351
**CLA+/CD4+** ^**§**^	72,81	81,77	0.164	0.477
**CD4 + CLA+/CD4+** ^**¥**^	42,57	49,17	0.618	0.405
**CD8 + CLA+** ^**§**^	7,856	4,908	0.089	0.591
**CLA+/CD8+** ^**§**^	78,09	58,85	0.420	0.271
**CD8 + CLA+/CD8+** ^**¥**^	37,17	20,39	0.767	0.550
**E-selectin** ^**§**^	1,217	539	0.892	0.046

^**¥**^ Student’s t-test; ^**§**^ Mann-Whitney Test; * p-value (p ≤ 0.05); GOLP: group of oral lichen planus; GORL: group of oral lichenoid reaction. “*d”* refers to effect size: no effect (*d* ≤ 0.1), small (0.20 ≤ *d* ≤ 0.40), medium (0.50 ≤ *d* ≤ 0.70), and large (*d* ≥ 0.80)

The average number of CLA + lymphocytes in the double immunofluorescence staining for CD4+/CLA + and CD8+/CLA + in both groups was not statistically significant (p = 0.840; d = 0.363).

The comparison of the number of CD4 + CLA + lymphocytes (p = 0.888; d = 0.351), as well as of CD8 + CLA + lymphocytes (p = 0.089; d = 0.591), was not statistically significant in both groups.

The proportion of CD4 + CLA + lymphocytes in relation to CD4 + lymphocytes in both groups was not statistically significant (p = 0.618; d = 0.405). Moreover, the proportion of CD8 + CLA + lymphocytes in relation to CD8 + lymphocytes (p = 0.767; d = 0.550); the proportion of CLA + lymphocytes in relation to CD4 + lymphocytes (p = 0.164; d = 0.477); and the proportion of CLA + lymphocytes in relation to CD8 + lymphocytes (p = 0.420; d = 0.271) was not statistically significant as well.

Considering immunostaining for E-selectin in each group, it was found 1,217 blood vessels in the GOLP and 539 in the GOLR. This difference was not statistically significant (p = 0.892; d = 0.046).

In GOLP, we performed an analysis to verify if there was a correlation between the number of CD4 + CLA + lymphocytes and E-selectin, but no correlation was found (p = 0.700). Likewise, we found no correlation for CD8 + CLA + lymphocytes and E-selectin (p = 0.951). In the GOLR, the same analysis was performed between CD4 + CLA + lymphocytes and E-selectin, but no correlation was verified (p = 0.454); and neither between CD8 + CLA + lymphocytes and E-selectin (p = 0.989).

## Discussion

OLR may be a delayed hypersensitivity reaction in which CD3+, CD4+, and CD8 + lymphocytes are involved [6, 7, 9, 10]. In all participants of the GOLR, we found the presence of CD3 + CD4+ (6,510, in total) and CD3 + CD8+ (5,918, in total) lymphocytes.

Although the difference between them was not statistically significant (p = 0.154), we know that these cells simultaneously act in the delayed hypersensitivity reaction. The antigen-presenting cells release cytokines that induce the proliferation of CD3 + CD4 + cells; at the same time, CD3 + CD8 + cells associated with the major histocompatibility complex (MHC) release cytokines that regulate late hypersensitivity reactions [7, 8].

The effect (d = 0.214) of the comparison between these lymphocyte populations was small, which means that the event is uncommon in the studied population. Therefore, these cells may not be the main ones in the process that culminates in the appearance of lichenoid reaction lesions, or they may act in association with other cells present in the inflammatory infiltrate such as B lymphocytes, plasma cells, mast cells, and eosinophils [22–24].

With regard to OLP, etiopathogenesis has not yet been fully elucidated, but it is known that CD3 + CD8 + lymphocytes play an important role [12, 25–28]. In the GOLP, the comparison between CD3 + CD4+ (15,678, in total) and CD3 + CD8+ (18,983, in total) lymphocytes resulted in a statistically significant difference (p < 0.001). This result corroborates the majority of studies previously published [28–31].

However, the effect of this comparison was small (0.353), which also demonstrates that other cells may have important roles in the etiopathogenesis of this disease. Authors, such as Matilla et al. [23] reported the presence of other cells, such as B lymphocytes, and other lymphocyte populations and, in some cases, these populations overlapped T lymphocytes. Werneck et al. [32] observed the presence of a greater number of CD3 + CD8 + cells when compared with CD3 + CD4 + cells in the OLP. The presence of a higher number of CD3 + CD8 + cells in OLP may be related to the etiopathogenesis of the disease, considering that the cytotoxic CD8 T lymphocytes, activated by auxiliary CD4 T lymphocytes, leading keratinocytes to apoptosis.

There was no statistical significance between the groups in the analysis of CD3 + CD4 + lymphocytes. Nevertheless, the presence of these cells has already been related to the patients’ age at the onset of the OLP lesion, i.e., those with lesions for longer periods of time would have more CD3 + CD4 + lymphocytes [25, 33]. When comparing the proportion of CD3 + CD4 + lymphocytes in relation to CD3 + lymphocytes, we noted a statistical significance between groups.

The comparison between groups regarding CD3 + CD8 + cells was not statistically significant, but the effect was medium (d = 0.571), which may indicate that the presence of these cells is relatively common in such lesions. The proportion of these cells was statistically significant, indicating that this relationship may be more important in OLP lesions than in ORL lesions.

When comparing the mean of lymphocytes per patient, we observed a slight predominance of CD3 + CD8 + lymphocytes in GOLP individuals, whereas in GOLR there is a slight predominance of CD3 + CD4 + lymphocytes. This difference was already expected according to the etiopathogenesis of the lesions, which has been reported by other authors [22, 28, 29].

The presence of CLA + T lymphocytes in the skin is well described in the literature, whether in diseases, such as lichen planus, or in other dermatological disorders [16, 17, 19, 29, 33, 34]. However, the oral mucosa is an area little explored in immunological studies, and there are few studies describing the presence of CLA + cells in OLP [22, 33]. Jang et al. [34] reported the presence of CLA + cells in cutaneous lichen planus lesions, and that these cells would not be found in cutaneous lichenoid lesions. We observed the presence of CLA + lymphocytes in both groups. However, the difference between them was not statistically significant (p = 0.840), and its effect was small (d = 0.363). Cutaneous lymphocyte-associated antigens are a lymphocyte subpopulation that can be expressed in Th1 and Th2 lymphocytes, cytotoxic T cells and regulatory T cells [34–36] of inflamed skin, oral mucosa, and normal skin [14, 15, 32]. Perhaps, this wide expression in several cells has made its event more common, hence characterizing a small effect. The average number of CLA + cells was higher in GOLR when compared with GOLP. Clark et al. [37] reported that squamous cell carcinoma lesions of the skin did not express E-selectin in the tumor areas and expressed few CLA + T lymphocytes, which was a curious fact, as it is believed that this lymphocyte is responsible for providing cutaneous immunosurveillance. Perhaps, this low number of CLA + cells is related to the potential for malignant transformation of OLP, which is not verified in lichenoid reactions. This aspect should be investigated in future research.

We observed differences in the CD4 + CLA + and CD8 + CLA + lymphocyte population in the GOLP (p = 0.031), with a small effect (d = 0.373). This was only previously reported by Werneck et al., [32] with no other studies demonstrating the presence of these lymphocyte populations. Sigmundsdóttir, [38] in psoriasis study, reported that CD3 + CD8 + CLA + cells were more related to the disease severity than CD3 + CD4 + CLA + cells. More studies are necessary to observe the degree of severity and/or the time of OLP lesion progression with the presence of these lymphocyte populations in order to confirm these data. However, we did not perform this type of analysis in the present study.

In the GOLR, we also found CD4 + CLA + and CD8 + CLA + lymphocytes; this difference was not statistically significant (p = 0.082), and the effect was small. This result may suggest that the difference between the number of these cells, within the same group, is not significant, but these cells may be relevant to the disease pathogenesis; or that lymphocytes which express CLA are not relevant to delayed hypersensitivity reactions, but rather to autoimmune inflammatory responses, as reported in the literature [38, 39].

We performed an intergroup analysis concerning the number of CD4 + CLA + lymphocytes and it was found 9,594 lymphocytes in the GOLP, and 4,954 in the GOLR. This difference was not statistically significant (p = 0.888), and the effect was small (d = 0.351). The same analysis was performed for CD8 + CLA + lymphocytes. We found 7,856 lymphocytes in the GOLP, and 4,908 in the GORL. Such difference was not statistically significant (p = 0.089); however, the effect was medium (d = 0.591). Although the number of lymphocytes in the GOLP is higher, the average number of CD4 + CLA + and CD8 + CLA + cells was higher in the GORL. The description of the presence of these cells is noteworthy, considering that there are very few studies in the oral mucosa. Furthermore, a higher average in lichenoid reaction lesions can be explained by the fact that the inflammatory infiltrate is band-like and deeper than that of the lichen planus lesion. A greater number of CD4 + CLA + lymphocytes in both groups may also be related to cell recruitment.

None of the proportions considered between the groups (CLA+/CD4+, CD4 + CLA+/CD4+, CLA+/CD8+, CD8 + CLA+/CD8+) was statistically significant, and the effects ranged from small to medium and the power was far from reaching 80%. According to this analysis, for better assess whether or not there is significance of these cells in the comparison between groups, a larger sample would be necessary. The presence of these cells is observed in both lesions, perhaps playing a secondary role in both recruiting and maintaining these lesions.

Finally, the correlation analysis between CD4 + CLA + lymphocytes and E-selectin, and between CD8 + CLA + lymphocytes and E-selectin was performed in both groups, but we found no significant correlation. In other studies on OLP and psoriatic patients, this relationship was not established as well [32, 40].

Although the analyzed correlation was not verified, and it will probably not be identified even with a larger sample, many of the investigations conducted in this study require a larger sample to be confirmed or contested. The analysis of the effect of each of the variables becomes important to assess the degree to which the event is present in a certain population in addition to the power analysis. Based on the results we observed that, for this study to reach the necessary power, a multicenter study would be necessary, as well as the evaluation of other adhesion molecules.

## Conclusion

CD3 + CD4+, CD3 + CD8+, CD4 + CLA+, CD8 + CLA + lymphocytes and E-selectin are present in both lesions. Nevertheless, only the proportion of CD3 + CD4 + and CD3 + CD8 + cells in relation to CD3 + cells is statistically significant, suggesting that these cells may be important in the etiopathogenic mechanism of OLP and OLR. The immunoexpression of E-selectin was not significant, and there was no correlation between CD4 + CLA+, CD8 + CLA + cells and E-selectin in the groups, suggesting that other adhesion molecules may participate in cell transmigration in the etiopathogenic mechanism of the lesions.

## Electronic supplementary material

Below is the link to the electronic supplementary material.

Supplementary Material 1

## Data Availability

The datasets used and/or analyzed during the current study are available from the corresponding author on reasonable request.

## List of abbreviations

**OLP**: Oral lichen planus.
**OLR**: Oral lichenoid reactions.
**MMP**: Metalloproteinase.
**RANTES**: Regulated upon activation, normal T cell expressed and secreted.
**TNF**: Tumor necrosis factor.
**CLA**: Cutaneous lymphocyte-associated antigens.
**GOLP**: Group of oral lichen planus.
**GOLR**: Group of oral lichenoid reaction.
**MHC**: Major histocompatibility complex.
