# Supplementary material for: Lymphocyte and CD62E expression in lichen planus and lichenoid reaction
Source: BMC Oral Health. 2022 Nov 17;22:507. doi: 10.1186/s12903-022-02496-5 (PMC9670570; doi:10.1186/s12903-022-02496-5)
Supplement: Supplementary file 1 — Supplementary Material 1 [file 12903_2022_2496_MOESM1_ESM.pdf]

Resultados das imunofluorescências de células CD3+, CD4+, CD8+, CLA+, e da selectina-E (CD62E) nos GLPO e GRLO de todos os participantes

GRUPO	Participante	Imuno	Número absoluto		Porcentagem	Imuno	Número absoluto		Porcentagem	Imuno	Número absoluto			Porcentagem		Imuno	Número absoluto			Porcentagem		Imuno	Número absoluto
			CD3+	CD3+CD4+	CD3+CD4+/CD3+		CD3+	CD3+CD8+	CD3+CD8+/CD3+		CD4+	CLA+	CD4+CLA+	CLA+/CD4+	CD4+CLA+/CD4+		CD8+	CLA+	CD8+CLA+	CLA+/CD8+	CD8+CLA+/CD8+		
Liquen Plano Oral (25)	#1	CD3/CD4	1428	938	65,68	CD3/CD8	1753	1199	68,39	CD4/CLA	1581	950	468	60,08	29,6	CD8/CLA	930	522	366	56,12	39,35	CD62E	85
	#2	CD3/CD4	775	608	78,45	CD3/CD8	1300	969	74,53	CD4/CLA	1441	727	554	50,41	38,44	CD8/CLA	1276	738	503	57,83	39,42	CD62E	56
	#3	CD3/CD4	945	674	71,32	CD3/CD8	1660	1430	86,14	CD4/CLA	1440	1022	425	70,97	29,51	CD8/CLA	1828	1176	620	64,33	33,91	CD62E	62
	#4	CD3/CD4	590	387	65,59	CD3/CD8	579	457	78,92	CD4/CLA	584	289	178	49,48	30,47	CD8/CLA	478	298	202	62,34	42,25	CD62E	41
	#5	CD3/CD4	743	631	84,92	CD3/CD8	1063	731	68,76	CD4/CLA	488	264	226	54,09	46,31	CD8/CLA	837	598	427	71,44	51,01	CD62E	34
	#6	CD3/CD4	831	584	70,27	CD3/CD8	873	771	88,31	CD4/CLA	805	539	280	66,95	34,78	CD8/CLA	559	397	359	71,01	64,22	CD62E	31
	#7	CD3/CD4	1012	855	84,48	CD3/CD8	1375	1272	92,5	CD4/CLA	1131	537	302	47,48	26,7	CD8/CLA	707	554	331	78,35	46,81	CD62E	32
	#8	CD3/CD4	172	86	50	CD3/CD8	92	75	81,52	CD4/CLA	343	213	60	62,09	17,49	CD8/CLA	148	337	48	227,7	32,43	CD62E	67
	#9	CD3/CD4	1362	1225	89,94	CD3/CD8	1906	1744	91,5	CD4/CLA	1068	716	428	67,04	40,07	CD8/CLA	984	646	181	65,65	18,39	CD62E	27
	#11	CD3/CD4	2185	1159	53,04	CD3/CD8	1334	950	71,21	CD4/CLA	1411	1193	594	84,54	42,42	CD8/CLA	1605	1114	582	69,4	36,26	CD62E	43
	#12	CD3/CD4	535	344	64,29	CD3/CD8	1249	696	55,72	CD4/CLA	846	375	143	44,32	16,9	CD8/CLA	1092	1112	483	101,83	44,23	CD62E	67
	#20	CD3/CD4	602	524	87,04	CD3/CD8	476	333	69,95	CD4/CLA	336	260	154	77,38	45,83	CD8/CLA	321	193	124	60,12	38,62	CD62E	37
	#37	CD3/CD4	535	253	47,28	CD3/CD8	1216	743	61,1	CD4/CLA	682	409	64	59,97	9,38	CD8/CLA	721	441	191	61,16	26,49	CD62E	32
	#38	CD3/CD4	522	249	47,7	CD3/CD8	796	515	64,69	CD4/CLA	1037	643	286	62	27,57	CD8/CLA	642	508	229	79,12	35,66	CD62E	59
	#40	CD3/CD4	1058	479	45,27	CD3/CD8	1190	564	47,39	CD4/CLA	789	353	260	44,74	32,95	CD8/CLA	660	311	216	47,12	32,72	CD62E	51
	#44	CD3/CD4	1159	625	53,92	CD3/CD8	3787	973	25,69	CD4/CLA	973	815	424	83,76	43,57	CD8/CLA	1183	946	489	79,96	41,33	CD62E	35
	#47	CD3/CD4	557	450	80,78	CD3/CD8	741	439	59,24	CD4/CLA	761	771	431	101,31	56,63	CD8/CLA	341	226	137	66,27	40,17	CD62E	53
	#48	CD3/CD4	875	745	85,14	CD3/CD8	678	475	70,05	CD4/CLA	1011	763	395	75,46	39,07	CD8/CLA	372	676	194	181,72	52,15	CD62E	28
	#49	CD3/CD4	894	695	77,74	CD3/CD8	1111	686	61,74	CD4/CLA	842	666	393	79,09	46,67	CD8/CLA	770	763	344	99,09	44,67	CD62E	104
	#50	CD3/CD4	1506	1305	86,65	CD3/CD8	1737	1417	81,57	CD4/CLA	1391	1286	744	92,45	53,48	CD8/CLA	1863	1000	399	53,67	21,41	CD62E	33
	#51	CD3/CD4	662	323	48,79	CD3/CD8	626	343	54,79	CD4/CLA	595	558	388	93,78	65,21	CD8/CLA	985	376	187	38,17	18,98	CD62E	45
	#54	CD3/CD4	854	586	68,61	CD3/CD8	1011	617	61,02	CD4/CLA	958	699	464	72,96	67,43	CD8/CLA	732	634	262	86,61	35,79	CD62E	52
	#55	CD3/CD4	1089	968	88,88	CD3/CD8	1036	647	62,45	CD4/CLA	1242	1244	968	100,16	77,93	CD8/CLA	1640	851	352	51,89	21,46	CD62E	44
	#56	CD3/CD4	745	569	76,37	CD3/CD8	678	394	58,11	CD4/CLA	746	922	612	123,59	82,03	CD8/CLA	780	636	465	81,53	59,61	CD62E	26
	#58	CD3/CD4	893	416	46,58	CD3/CD8	868	543	62,55	CD4/CLA	552	532	353	96,37	63,94	CD8/CLA	1385	553	165	39,92	11,91	CD62E	73
		**TOTAL**	**22529**	**15678**		**TOTAL**	**29135**	**18983**		**TOTAL**	**23053**	**16746**	**9594**			**TOTAL**	**22839**	**15606**	**7856**			**TOTAL**	**1217**
		**MÉDIA**	**901,16**	**627,12**	**68,74**	**MÉDIA**	**1165,4**	**759,32**	**67,91**	**MÉDIA**	**922,12**	**669,84**	**383,76**	**72,81**	**42,57**	**MÉDIA**	**913,56**	**624,2**	**314,24**	**78,09**	**37,17**	**MÉDIA**	**48,68**
Reação Liquenóide (11)	#15	CD3/CD4	2414	529	21,91	CD3/CD8	2629	421	16,01	CD4/CLA	1104	1025	643	92,84	58,24	CD8/CLA	1507	1670	885	110,81	58,72	CD62E	50
	#17	CD3/CD4	825	506	61,33	CD3/CD8	1057	536	50,7	CD4/CLA	847	749	470	88,42	55,48	CD8/CLA	1267	889	447	70,16	35,28	CD62E	37
	#22	CD3/CD4	1373	848	61,76	CD3/CD8	873	724	82,93	CD4/CLA	1023	1043	745	101,95	72,82	CD8/CLA	789	604	411	76,55	52,09	CD62E	42
	#34	CD3/CD4	1106	531	48,01	CD3/CD8	1046	650	62,14	CD4/CLA	979	871	534	88,96	54,54	CD8/CLA	992	785	501	79,13	50,5	CD62E	50
	#39	CD3/CD4	2837	926	32,64	CD3/CD8	2042	472	23,11	CD4/CLA	1266	538	267	42,49	21,09	CD8/CLA	1193	709	501	59,43	41,99	CD62E	35
	#41	CD3/CD4	586	471	80,37	CD3/CD8	469	327	69,72	CD4/CLA	607	529	380	87,14	62,6	CD8/CLA	943	744	510	78,89	54,08	CD62E	24
	#43	CD3/CD4	571	204	35,72	CD3/CD8	1324	264	19,93	CD4/CLA	354	330	156	93,22	44,06	CD8/CLA	288	315	107	109,37	37,15	CD62E	63
	#45	CD3/CD4	327	212	64,83	CD3/CD8	509	219	43,02	CD4/CLA	620	448	240	72,25	38,7	CD8/CLA	481	401	223	83,36	46,36	CD62E	34
	#52	CD3/CD4	1516	1012	66,75	CD3/CD8	894	492	55,03	CD4/CLA	1262	634	402	50,23	31,85	CD8/CLA	928	455	319	49,03	34,37	CD62E	42
	#53	CD3/CD4	1059	673	63,55	CD3/CD8	1391	844	60,67	CD4/CLA	1363	1310	559	96,11	41,01	CD8/CLA	1611	1093	776	67,84	48,16	CD62E	78
	#57	CD3/CD4	962	598	62,16	CD3/CD8	1128	969	85,9	CD4/CLA	922	792	558	85,9	60,52	CD8/CLA	1118	658	228	58,85	20,39	CD62E	84
		**TOTAL**	**13576**	**6510**		**TOTAL**	**13362**	**5918**		**TOTAL**	**10347**	**8269**	**4954**			**TOTAL**	**11117**	**8323**	**4908**			**TOTAL**	**539**
		**MÉDIA**	**1234,2**	**591,81**	**54,45**	**MÉDIA**	**1214,7**	**538**	**51,74**	**MÉDIA**	**940,63**	**751,72**	**450,36**	**81,77**	**49,17**	**MÉDIA**	**1010,63**	**756,6**	**446,18**	**76,67**	**43,55**	**MÉDIA**	**49**

Imuno= Imunofluorescência

Resultados das médias de células CD3+ e CLA+ nos GLPO e GRLO de todos os participantes

	Participante	CD3 +(CD3+/CD4+)	CD3 +(CD3+/CD8+)	MÉDIA	CLA+(CD4+/CLA+)	CLA+(CD8+/CLA+)	MÉDIA
**Liquen Plano Oral (25)**	#1	1428	1753	1590,5	950	522	736
	#2	775	1300	1037,5	727	738	732,5
	#3	945	1660	1302,5	1022	1176	1099
	#4	590	579	584,5	289	298	293,5
	#5	743	1063	903	264	598	431
	#6	831	873	852	539	397	468
	#7	1012	1375	1193,5	537	554	545,5
	#8	172	92	132	213	337	275
	#9	1362	1906	1634	716	646	681
	#11	2185	1334	1759,5	1193	1114	1153,5
	#12	535	1249	892	375	1112	743,5
	#20	602	476	539	260	193	226,5
	#37	535	1216	875,5	409	441	425
	#38	522	796	659	643	508	575,5
	#40	1058	1190	1124	353	311	332
	#44	1159	3787	2473	815	946	880,5
	#47	557	741	649	771	226	498,5
	#48	875	678	776,5	763	676	719,5
	#49	894	1111	1002,5	666	763	714,5
	#50	1506	1737	1621,5	1286	1000	1143
	#51	662	626	644	558	376	467
	#54	854	1011	932,5	699	634	666,5
	#55	1089	1036	1062,5	1244	851	1047,5
	#56	745	678	711,5	922	636	779
	#58	893	868	880,5	532	553	542,5
	**TOTAL**	**22529**	**29135**	**25832**	**16746**	**15606**	**16176**
**Reação Liquenóide (11)**	#15	2414	2629	2521,5	1025	1670	1347,5
	#17	825	1057	941	749	889	819
	#22	1373	873	1123	1043	604	823,5
	#34	1106	1046	1076	871	785	828
	#39	2837	2042	2439,5	538	709	623,5
	#41	586	469	527,5	529	744	636,5
	#43	571	1324	947,5	330	315	322,5
	#45	327	509	418	448	401	424,5
	#52	1516	894	1205	634	455	544,5
	#53	1059	1391	1225	1310	1093	1201,5
	#57	962	1128	1045	792	658	725
	**TOTAL**	**13576**	**13362**	**13469**	**8269**	**8323**	**8296**
